# Deterioration of anterior resin composite restorations in moderate to severe tooth wear patients: 3-year results

**DOI:** 10.1007/s00784-022-04647-y

**Published:** 2022-07-26

**Authors:** Verônica P. Lima, Luuk A. M. J. Crins, Niek J. M. Opdam, Rafael R. Moraes, Ewald M. Bronkhorst, Marie-Charlotte D. N. J. M. Huysmans, Bas A. C. Loomans

**Affiliations:** 1 Graduate Program in Dentistry, Federal University of Pelotas, Rua Gonçalves Chaves 457, Pelotas, RS 96015-560 Brazil; 2 Radboud Institute for Health Sciences, Department of Dentistry, Radboud University Medical Center, Ph. van Leydenlaan 25, NL 6500 HB, P.O. Box 9101, Nijmegen, 6525 EX The Netherlands

**Keywords:** Anterior resin composite restorations, Restorations, Deterioration

## Abstract

**Objectives:**

Deterioration in anterior resin composite restorations placed in tooth wear patients was investigated after 36 months.

**Materials and methods:**

Data collected prospectively for 47 participants of the Radboud Tooth Wear Project were used (41 ± 8 years, 90% male, *n* = 270 restorations). Restorations were individually evaluated using intraoral photographs and 3D scans to rate modified FDI scores and to record the presence of degradation features. Four groups with distinct combinations of composites and techniques were assessed, and multivariable logistic regression models were used to analyze the data (*p* < 0.05).

**Results:**

For all groups together, early degradation signs were present at 1 month: irregularities (41.5%) and ditching (7.4%) were observed at the surface and adhesive interfaces. The frequency of irregularities decreased in the 36-month evaluation (37%), but ditching (12.2%) and fractures (10.7%) were more common. The most frequent deterioration (based on photographs) was observed for staining (44%) and loss of luster (31%). In 3D scans, the most frequent were for wear (25%), marginal adaptation (24%), and the presence of irregularities (19%). Canines had 5.5 times more chances of deterioration by ditching than incisors (*p* < 0.001). The differences between composites and restorative techniques were minor.

**Conclusions:**

A continuous degradation process of restorations placed in tooth wear patients was observed in anterior teeth restored with different composites, with a progression of the deterioration over 36 months.

**Clinical relevance:**

When placing anterior resin composite restorations in tooth wear patients, it could be important to establish realistic expectations and the need for checkup appointments.

## Introduction

The nature and number of teeth affected might impact the challenges imposed on restorative treatments. The etiological factors that caused the tooth wear and high masticatory loading will most likely still be present after any restorative treatment is performed, with repercussions in the short and long terms. Varied direct and indirect techniques to reconstruct the worn dentition and reestablish vertical dimension of occlusion (VDO) using resin composite have been reported [1–8].

The deterioration process of composite restorations in patients with severe tooth wear may include the development of wear facets, marginal degradation, surface and interface irregularities, staining, discoloration, chipping, and bulk fractures [1–9]. A peculiarity of restorations in severe tooth wear patients seems to be the shorter time for signs of deterioration to be detectable compared with patients without the condition. A clinical trial [1] on direct composites placed on worn mandibular anterior teeth found wear facets in 10% of restorations in the first few months of their clinical service. Longevity data are available for the behavior of anterior composite restorations in tooth wear patients [2, 3, 7, 8, 10]. However, the literature lacks evidence on the nature and severity of degradation or adverse events taking place at the restorative materials and bonded interfaces during clinical service.

The interfaces between materials or between material and tooth are regarded as weak links in adhesive restorations [11, 12], especially when the margins involve dentin/cementum. In terms of mechanical stability, the matter may comprehend differences in elastic properties, stress concentration and dissipation, endurance limits, and time-dependent behaviors between the distinct components forming the restorative interfaces. A recent clinical trial evaluating composite restorations with an interface between two composite layers found chip fractures as the most common reason for failure after 3.5 years [7]. A fourfold failure rate for anterior restorations placed in two clinical sessions compared with single-session restorations also was observed. These findings draw attention to the early deterioration in anterior restorations when an interface between composites is present. However, the available evidence on restorations placed in worn anterior dentition is scarce [4, 7]. Understanding the degradation taking place at restorative materials and interfaces during the clinical service could aid in developing longer-lasting restorative protocols for severe tooth wear patients.

The aim of this study was to investigate the deterioration in anterior resin composite restorations placed in patients with moderate to severe tooth wear after 3 years of clinical service. The restorations were carried out with varied types of composites and techniques. Photographic images and 3D scans collected after 1 and 36 months of follow-up were used to identify “degradation features” present on restorative materials and interfaces. The hypothesis was that a deterioration process would be detectable in restorations after 3 years.

## Materials and methods

This article reports a longitudinal analysis involving participants from the Radboud Tooth Wear Project (RTWP) [13], which is a project that includes multiple prospective clinical studies on the treatment of patients with moderate to severe tooth wear at the Department of Dentistry of the Radboud University Medical Center, Nijmegen, The Netherlands. For each clinical study, ethical approval was sought and granted before the study was undertaken (CMO Arnhem-Nijmegen: NL30346.091.10, NL31371.091.10 and 2014–1252). The present study was carried out in accordance with the Declaration of Helsinki for research involving humans.

### Study design

This non-controlled clinical study uses data collected prospectively from patients participating in the RTWP [13] who had been restoratively treated between 2011 and 2018. All patients who had anterior maxillary teeth restorations placed in the period were eligible. Participants returned for follow-up appointments 1 and 36 months after completion of the restorative rehabilitation. At each follow-up appointment, intraoral photographs and 3D scans were acquired, and these data were used in the present investigation. The anterior restorations were assessed individually to investigate deterioration by using modified FDI criteria for evaluating restorations [14], which involve judging several aspects of the restorations with scores from 1 (clinically excellent) up to 5 (clinically poor). Additionally, the presence of “degradation features” in the restorations, namely ditching, irregularities, and small fractures, was classified as absent or present. The primary outcome was the occurrence of deterioration after 36 months of follow-up: any aspect of deterioration was considered present when there was an increase in FDI scores after 36 months or when a “degradation feature” was absent at 1 month but present at 36 months.

### Participants, inclusion, and exclusion criteria

Patients were referred by their general dental practitioners to the RTWP. Inclusion criteria were (1) age ≥ 18 years; (2) good general health; (3) moderate to severe generalized tooth wear (tooth wear index: mean max TWI-score ≥ 2: loss of enamel exposing dentin for less than one third of the surface) [15]; and (4) full dental arches with a maximum of one missing posterior tooth. Exclusion criteria were (1) local or systemic conditions that would contraindicate dental procedures; (2) temporomandibular disorders; (3) deep caries lesions or endodontic problems; and (4) advanced periodontitis. Specific individual etiological factors for tooth wear were not considered as exclusion criteria, including parafunctional habits such as grinding, clenching, and gastroesophageal reflux disease. To be included in this convenience sample, patients had to present both buccal and palatal surfaces of anterior maxillary teeth restored with resin composite (palatal and buccal veneers). Patients were excluded if 3D scans and/or photographs were unavailable for the 1 and/or 36-month time points or if presenting more than one combination of materials, e.g., incisors and canines restored with different materials.

### Clinical and restorative procedures

At the first appointment, the patients signed a written informed consent, and a full intraoral examination was carried out, including intraoral photographs. Patients were allocated to one of 7 operators to receive a full mouth rehabilitation using resin composite restorations, including an increase of the VDO [13, 16]. The intended increase of VDO was estimated by two operators using stone cast models, based on the height loss of the teeth that had the most amount of tooth wear. One of four different restorative protocols that combined different direct and/or indirect composites was used to reconstruct the worn anterior maxillary teeth. All materials used, surface treatments, and restorative clinical procedures are detailed in Table 1. The decision on which protocol to use was based on the necessary increase of the VDO: patients in need of an increase of 3 mm or less received direct composite restorations; if the need was more than 3 mm, they received a combination of indirect and direct restorations. Intraoral mockups on anterior maxillary teeth, from canine to canine, were made to assess the possibility to lengthen these teeth and check the intended increase in VDO. Posterior teeth were always restored with direct composite. Complete rehabilitations required between three and five clinical sessions of approximately 3 h each, with an interval of one to two weeks between appointments [7, 16].

**Table 1:** Restorative materials and procedures used to reconstruct the anterior teeth with severe tooth wear

Technique for palatal veneer	Clinical sessions^a^	Code	Composite used in palatal veneer	Composite used in buccal veneer	Other materials used^b^	Surface treatments and procedures
Direct composite restorations	1 or 2	APX + IPS	Clearfil AP-X (Kuraray Noritake, Japan)	IPS Empress Direct (Ivoclar Vivadent, Liechtenstein)	Etchant – 37% phosphoric acid (DMG, Germany); Three-step etch-and-rinse adhesive system – Clearfil SA primer and Clearfil Photobond (Kuraray Noritake); Light curing unit – Bluephase 16i, maximum irradiance 1600 mW/cm^2^ (Ivoclar Vivadent); Metal matrix – Tofflemire n.11 (KerrHawe Sa, Switzerland); Plastic matrix – Directa Matrix Strips (Directa, Sweden)	Phosphoric acid was applied for 15–30 s, rinsed thoroughly, and dried with air. Primer was applied to the dental surface and evaporated. The bonding agent was applied, the solvent evaporated and light-cured for 10 s. Metal and plastic matrices were used for gingival contouring. The restorations were placed according to the DSO-technique [9], starting by the palatal veneer by the buccal veneer. Buccal veneers were placed in the same or in a subsequent session [9, 14]. The restorations were finished with fine grit diamond burs and polishing rubbers
		SUP + SUP	Filtek Supreme XTE (3 M, USA)	Filtek Supreme XTE (3 M)		
Indirect composite restorations	2^c^	EST + IPS	Clearfil Estenia C&B (Kuraray Noritake)	IPS Empress Direct (Ivoclar Vivadent)	30-μm silica-coated alumina particles – CoJet System (3 M); Etchant – 37% phosphoric acid (DMG); Bonding agent – Clearfil Ceramic Primer (Kuraray Noritake); Dual-cured resin cement – Panavia F (Kuraray Noritake)	Palatal veneers were made in increments in the dental lab (light- and heat-cured). Restorations were sandblasted with 50-µm alumina particles. The veneer was checked for its seating, the surface was cleaned with phosphoric acid. Silane was applied. Surface of the abutment tooth was air-abraded with 30-µm CoJet particles in case of pre-existing composite restoration. The tooth surface was etched with phosphoric acid and rinsed. A layer of the cement primer and a layer of the resin cement were applied on the palatal veneer and placed on the tooth. The cement was light-cured for 1–2 s, the excess removed, then each surface was light-cured for 20 s. The occlusion was checked. The restorations were finished with fine grit diamond burs and polishing rubbers. Before placement of the buccal veneers, the palatal veneers were air-abraded with 30-µm CoJet particles and silane was applied. Direct buccal veneer restorations were placed in a subsequent session, as previously described [14]
		LU + SUP	Lava Ultimate (3 M)	Filtek Supreme XTE (3 M)	30 μm silica-coated alumina particles (CoJet); Bonding agent – ESPESIL (3 M) Adhesive system – Scotchbond Universal (3 M); Etchant – Scotchbond Universal Etchant 37% phosphoric acid (3 M); Dual-cured resin cement – RelyX Ultimate (3 M)	Palatal veneers were milled and polished in a dental lab. The veneer was checked for its seating, the surface was cleaned and roughened using air-abrasion. A layer of silane and a layer of adhesive were applied. The surface of the abutment tooth was air-abraded with 30-µm CoJet particles in case of pre-existing composite restoration. The dental surface was etched with phosphoric acid and rinsed. A layer of adhesive was applied to the dental surface. A layer of the resin cement was applied on the veneer and placed on the tooth. After light-curing it for 1–2 s, excess of cement was removed, then each surface was light-cured for 20 s. The occlusion was checked. The restorations were finished with fine grit diamond burs and polishing rubbers. Before placement of the buccal veneers, the palatal veneers were air-abraded with 30-µm CoJet particles and silane was applied. Direct buccal veneer restorations were placed in a subsequent session, as previously described [14]

^a^Refers to the moment of placement of the buccal veneer: 1 session, placed on the same day as the palatal veneer; 2 sessions, placement on different days

^b^All materials were used according to manufacturers’ directions

^c^The palatal veneer was made of indirect composite, thus requiring roughening of the surface with air-abrasion before placement of the buccal veneer. For that reason, even if the buccal veneer’s placement was done in 1 session, the palatal veneer was regarded as an ‘old’ substrate; thus, the placement was considered as done in 2 sessions

The palatal surface of the anterior teeth was made of direct or indirect resin composite. When direct composite was used, either the microhybrid Clearfil AP-X or the nanofilled composite Filtek Supreme XTE were used for the palatal veneers. These two composites have differences in filler loading and particle morphology, but both are indicated for stress-bearing areas. When the palatal veneer was made indirectly, Clearfil Estenia C&B or Lava Ultimate were the indirect composites. Direct resin composite was always applied as veneering material at the buccal surfaces: the nanohybrid IPS Empress Direct was used when the palatal veneer was APX (APX-IPS) or Estenia (EST-IPS), whereas Filtek Supreme XTE was used when the palatal veneer was made of Filtek Supreme XTE (SUP-SUP) or Lava Ultimate (LU + SUP). Direct restorations were made using the Direct Shaping by Occlusion technique [16]. The intermaxillary space was registered using polyvinyl siloxane stops (Star VPS, Danville Materials, USA) placed bilaterally in the posterior area. The bite stops functioned as a guide to reach the intended VDO and to ensure sufficient intermaxillary space for the anterior teeth. The posterior teeth were built-up using the same direct composite used in the palatal veneer of the anterior restorations (APX or SUP).

### Evaluation of restorations

One and thirty-six months after placing the restorations, the patients returned for follow-up appointments. At each follow-up time, new intraoral photographs were taken (Camera EOS 70D, Canon, manual, shutter 1:100, aperture 22; Lens F017 Macro-objective 90 mm, Tamron; Flash MR14-EX Macro Ring Lite, Canon) and 3D scans acquired (Lava™ Chairside Oral Scanner C.O.S. and 3 M™ True Definition Intraoral Scanner, 3 M). After 3D scans acquisition, the files were stored in the web-based platform Casemanager (3 M), downloaded from this platform as STL-files, and then imported to the software MeshLab [17] where the models could be rotated, zoomed in and out for assessment. The scans were assessed as acquired, with no further processing or refining of the images. One experienced and trained examiner evaluated all anterior restorations by assessing the intraoral photographs and 3D scans. A second examiner evaluated a random sample of 72 restorations from 13 patients to check reproducibility of the photographic and 3D scan analyses; inter- and intra- examiners agreements were determined. The examiners were blind to the combination of materials and follow-up time. The photographs and 3D scans were assessed independently, anonymously, and in random order with no comparison between the baselines and follow-up. Modified FDI criteria [14] were adopted, as detailed in Table 2, considering esthetic, functional, and biological properties. The modifications were carried out because of the severity of tooth wear in this group of patients. In functional properties, the criterion “occlusal contour and wear” was altered to “incisal contour and wear” since only anterior restorations were considered. The descriptions of the scores for “incisal contour and wear” were adjusted to consider the presence of wear facets. In biological properties, abfraction was not considered separately in “recurrence of erosive tooth wear/caries.” The esthetic properties criteria were rated using only the intraoral photographs and no modifications were made to these criteria. Functional and biological properties were rated by using only the 3D scans. The presence or absence of “degradation features” at the interfaces (irregularities, ditching, or small fractures) was an additional evaluation assessed using only the 3D scans and aided in obtaining qualitative data on the deterioration process.

**Table 2:** Modified FDI criteria [15] used for evaluating the restorations

	Esthetic properties				Functional properties			Biological properties	
	*Surface luster*	*Staining*	*Color match and translucency*	*Esthetic anatomical form*	*Fracture of material and retention*	*Marginal adaptation*	*Incisal contour and wear*	*Recurrence of erosive tooth wear/caries*	*Tooth integrity*
1. Clinically excellent/ very good	Luster comparable to enamel	No staining	Good color match, no difference in shade and/or translucency	Form is ideal	No fractures/cracks	Harmonious outline, no gaps, no white or discolored lines	Physiological wear, no visible wear facets	No erosive tooth wear No secondary or primary caries	Complete integrity
2. Clinically good	Slightly dull, not noticeable from speaking distance; Some isolated pores	Minor staining, easily removable by polishing	Minor deviations in shade and/or translucency	Form is only slightly deviated from the normal	Small hairline crack	Marginal gap (< 150 μm), white lines; Small marginal fracture removable by polishing; Slight ditching, slight step/flashes, minor irregularities	Normal wear, presence of small facets	Small and localized erosive tooth wear, demineralization	Small marginal enamel fracture (< 150 μm)
3. Clinically sufficient/ satisfactory	Dull surface but acceptable if covered with film of saliva; Multiple pores on more than one third of the surface	Moderate staining that may also present on other teeth, not esthetically unacceptable	Distinct deviation but acceptable. Does not affect esthetics	Form deviates from the normal but is esthetically acceptable	Two or more large hairline cracks and/or material chip fracture not affecting the marginal integrity or approximal contact	Gap < 250 μm not removable; Several small marginal fractures; Major irregularities, ditching or flash, steps	Significant wear, larger facets	Larger areas of erosive tooth wear, demineralization Dentin not exposed	Hairline crack in enamel (< 150 μm)
4. Clinically unsatisfactory (but repairable)	Rough surface cannot be masked by saliva film, simple polishing is not sufficient; Further intervention necessary; Voids	Unacceptable staining on the restoration and major intervention necessary for improvement	Localized clinically deviation that can be corrected by repair	Form is affected and unacceptable esthetically. Intervention/correction is necessary	Material chip fractures which damage marginal quality or approximal contacts Bulk fractures with partial loss (< 1/2 of the restoration)	Gap > 250 μm or dentine/base exposed; Severe ditching or marginal fractures; Larger irregularities or steps (repair necessary)	Considerable wear, restoration partially lost (up to 1/3). The underlying dental surface is visible but intact	Erosive tooth wear in dentin. Caries with cavitation and suspected undermining caries	Marginal enamel defect < 250 μm Crack < 250 μm Enamel chipping. Multiple cracks
5. Clinically poor (replacement necessary)	Very rough, unacceptable plaque retentive surface	Severe staining, generalized or localized, not accessible for intervention	Unacceptable. Replacement necessary	Form is unsatisfactory and/or lost. Repair not feasible / reasonable, Replacement needed	Partial or complete loss of restoration or multiple fractures	Restoration (complete or partial) is loose but in situ; Generalized major gaps or irregularities	Wear is excessive, restoration completely lost. Wear of the underlying dental surface	Excessive erosive tooth wear in dentin (> 2 mm width). Deep caries or exposed dentine that is not accessible for repair of restoration	Cusp or tooth fracture

### Data analysis

Cohen’s kappa coefficient for the inter-examiner (0.60–0.94) and intra-examiner agreements (0.66–0.98) were moderate to good and substantial to good, respectively. Thus, evaluations made by the first examiner were adopted. Frequencies tables were used to describe the distributions of the FDI criteria and the presence of “degradation features” after 1 and 36 months of clinical service. “Degradation features” were considered as the presence of ditching, irregularities, and small fractures. Statistical analysis was performed separately for each evaluation criterion. Since the buccal veneers were made with only two direct composites (IPS or SUP), groups with similar veneering materials (APX + IPS and EST + IPS; SUP + SUP and LU + SUP) were analyzed together in the comparisons of esthetic properties. The occurrence of deterioration for each criterion/degradation feature was analyzed using multilevel multivariable logistic regression models with random intercepts to adjust for clustering by patient. The major outcome — the deterioration — was considered present when there was increase in FDI scores after 36 months or when a “degradation feature” was absent at 1 month but present at 36 months. The dependent variables, i.e., the criteria and features assessed, were adopted as clinical parameters to determine the degradation. All dependent variables were categorical (yes/no) and attributed “yes” in case of the presence of deterioration and “no” in its absence. Since anterior restorations were evaluated, only two groups of teeth were assessed (incisors and canines), and this variable was considered in the regression model. The independent variables were the type of tooth (coded as “0” if the tooth was incisor, “1” if was canine) and combination of restorative materials (APX + IPS, EST + IPS, SUP + SUP, LU + SUP), with APX + IPS as the reference type. In all statistical tests, a 5% confidence level was adopted. Statistical analyses were carried out using R v.3.6.3 (R Core Team, 2019) [18].

## Results

From the 117 eligible patients in the RTWP, intraoral photographs or 3D scans were missing in at least one follow-up (1 or 36 months) for 45 patients, and 25 patients had anterior maxillary restorations made with more than one combination of restorative materials. Thus, the present sample included 47 patients with a total of 270 anterior resin composite restorations; the mean age was 43 years (range 32–61), and 90% were male. The participants’ overall mean maximum TWI-scores before treatment had a mean ± standard deviation (SD) of 2.4 ± 0.4. Regarding the combination of restorative materials, 17 patients were restored with a combination of APX + IPS (102 restorations), 8 with EST + IPS (46 restorations), 15 with SUP + SUP (80 restorations), and 7 with LU + SUP (42 restorations). The mean ± SD increase of VDO measured at the first molars after treatment was 2.0 ± 0.9 mm.

Table 3 shows the frequency of scores attributed to restorations according to modified FDI criteria [14]. For esthetic properties, at 1 month, the highest score observed was 3, whereas restorations were rated with scores 4 at 36 months for staining (1.1%), surface luster (0.7%), and esthetic anatomical form (2.2%). Surface luster also was rated with score 5 at 36 months (0.7%). For functional properties, in corroboration with the 1-month evaluation, the highest score rated was 3. For the 36-month evaluation, scores 4 were rated for fracture of material and retention (2.6%) and marginal adaptation (2.2%). Tooth integrity showed only scores 1 at both follow-up times. Recurrence of erosive tooth wear/caries showed 99% scores 1 in the first analysis and 97% scores 1 after 36 months.

**Table 3:** Frequency and percentage of FDI scores attributed to restorations after 1 and 36 months of follow-up according to the combination of restorative materials (*N* = 47 patients, number of restorations in each group shown in parenthesis)

FDI criterion	Score	1 month								36 months							
		APX + IPS (n = 102)		EST + IPS (n = 46)		SUP + SUP (n = 80)		LU + SUP (n = 42)		APX + IPS (n = 102)		EST + IPS (n = 46)		SUP + SUP (n = 80)		LU + SUP (n = 42)	
		Freq	%	Freq	%	Freq	%	Freq	%	Freq	%	Freq	%	Freq	%	Freq	%
*Surface luster*	1	68	66.7	31	67.4	51	63.8	36	85.7	54	52.9	16	34.8	48	60	28	66.7
	2	34	33.3	15	32.6	29	36.3	6	14.3	41	40.2	24	52.2	27	33.8	7	16.7
	3	0	0	0	0	0	0	0	0	7	6.9	4	8.7	3	3.8	7	16.7
	4	0	0	0	0	0	0	0	0	0	0	2	4.3	0	0	0	0
	5	0	0	0	0	0	0	0	0	0	0	0	0	2	2.5	0	0
*Staining*	1	96	94.1	40	87	74	92.5	36	85.7	56	54.9	20	43.5	37	46.3	21	50
	2	6	5.9	6	13	6	7.5	6	14.3	37	36.3	21	45.7	35	43.8	20	47.6
	3	0	0	0	0	0	0	0	0	9	8.8	4	8.7	6	7.5	1	2.4
	4	0	0	0	0	0	0	0	0	0	0	1	2.2	2	2.5	0	0
	5	0	0	0	0	0	0	0	0	0	0	0	0	0	0	0	0
*Color match and translucency*	1	90	88.2	45	97.8	75	93.8	34	81	65	63.7	36	78.3	74	92.5	36	85.7
	2	12	11.8	1	2.2	4	5	7	16.7	33	32.4	9	19.6	5	6.3	6	14.3
	3	0	0	0	0	1	1.3	1	2.4	4	3.9	1	2.2	1	1.3	0	0
	4	0	0	0	0	0	0	0	0	0	0	0	0	0	0	0	0
	5	0	0	0	0	0	0	0	0	0	0	0	0	0	0	0	0
*Esthetic anatomical form*	1	98	96.1	41	89.1	73	91.3	40	95.2	91	89.2	40	87	67	83.8	39	92.9
	2	4	3.9	4	8.7	5	6.3	2	4.8	7	6.9	4	8.7	8	10	3	7.1
	3	0	0	1	2.2	2	2.5	0	0	3	2.9	0	0	2	2.5	0	0
	4	0	0	0	0	0	0	0	0	1	1	2	4.3	3	3.8	0	0
	5	0	0	0	0	0	0	0	0	0	0	0	0	0	0	0	0
*Fracture of material and retention*	1	99	97.1	44	95.7	76	95	40	95.2	93	91.2	38	82.6	69	86.3	36	85.7
	2	0	0	0	0	0	0	0	0	0	0	0	0	2	2.5	1	2.4
	3	3	2.9	2	4.3	4	5	2	4.8	8	7.8	5	10.9	6	7.5	5	11.9
	4	0	0	0	0	0	0	0	0	1	1	3	6.5	3	3.8	0	0
	5	0	0	0	0	0	0	0	0	0	0	0	0	0	0	0	0
*Marginal adaptation*	1	50	49	20	43.5	43	53.8	26	61.9	49	48	20	43.5	33	41.3	23	54.8
	2	47	46.1	25	54.3	33	41.3	15	35.7	46	45.1	19	41.3	36	45	15	35.7
	3	5	4.9	1	2.2	4	5	1	2.4	6	5.9	5	10.9	8	10	4	9.5
	4	0	0	0	0	0	0	0	0	1	1	2	4.3	3	3.8	0	0
	5	0	0	0	0	0	0	0	0	0	0	0	0	0	0	0	0
*Incisal contour and wear*	1	48	47.1	32	69.6	31	38.8	39	92.9	41	40.2	38	82.6	41	51.3	28	66.7
	2	43	42.2	14	30.4	37	46.3	3	7.1	42	41.2	7	15.2	32	40	13	31
	3	11	10.8	0	0	12	15	0	0	19	18.6	1	2.2	7	8.8	1	2.4
	4	0	0	0	0	0	0	0	0	0	0	0	0	0	0	0	0
	5	0	0	0	0	0	0	0	0	0	0	0	0	0	0	0	0
*Recurrence of erosive tooth wear/ caries*	1	100	98	46	100	80	100	42	100	100	98	42	91.3	78	97.5	42	100
	2	2	2	0	0	0	0	0	0	2	2	2	4.3	2	2.5	0	0
	3	0	0	0	0	0	0	0	0	0	0	2	4.3	0	0	0	0
	4	0	0	0	0	0	0	0	0	0	0	0	0	0	0	0	0
	5	0	0	0	0	0	0	0	0	0	0	0	0	0	0	0	0
*Tooth integrity*	1	102	100	46	100	80	100	42	100	102	100	46	100	80	100	42	100
	2	0	0	0	0	0	0	0	0	0	0	0	0	0	0	0	0
	3	0	0	0	0	0	0	0	0	0	0	0	0	0	0	0	0
	4	0	0	0	0	0	0	0	0	0	0	0	0	0	0	0	0
	5	0	0	0	0	0	0	0	0	0	0	0	0	0	0	0	0

APX + IPS (palatal veneer Clearfil AP-X, buccal veneer IPS Empress Direct), EST + IPS (palatal veneer Clearfil Estenia C&B, buccal veneer IPS Empress Direct), SUP + SUP (palatal and buccal veneers Filtek Supreme XTE), LU + SUP (palatal veneer Lava Ultimate, buccal veneer Filtek Supreme XTE)

**Fig. 1:**
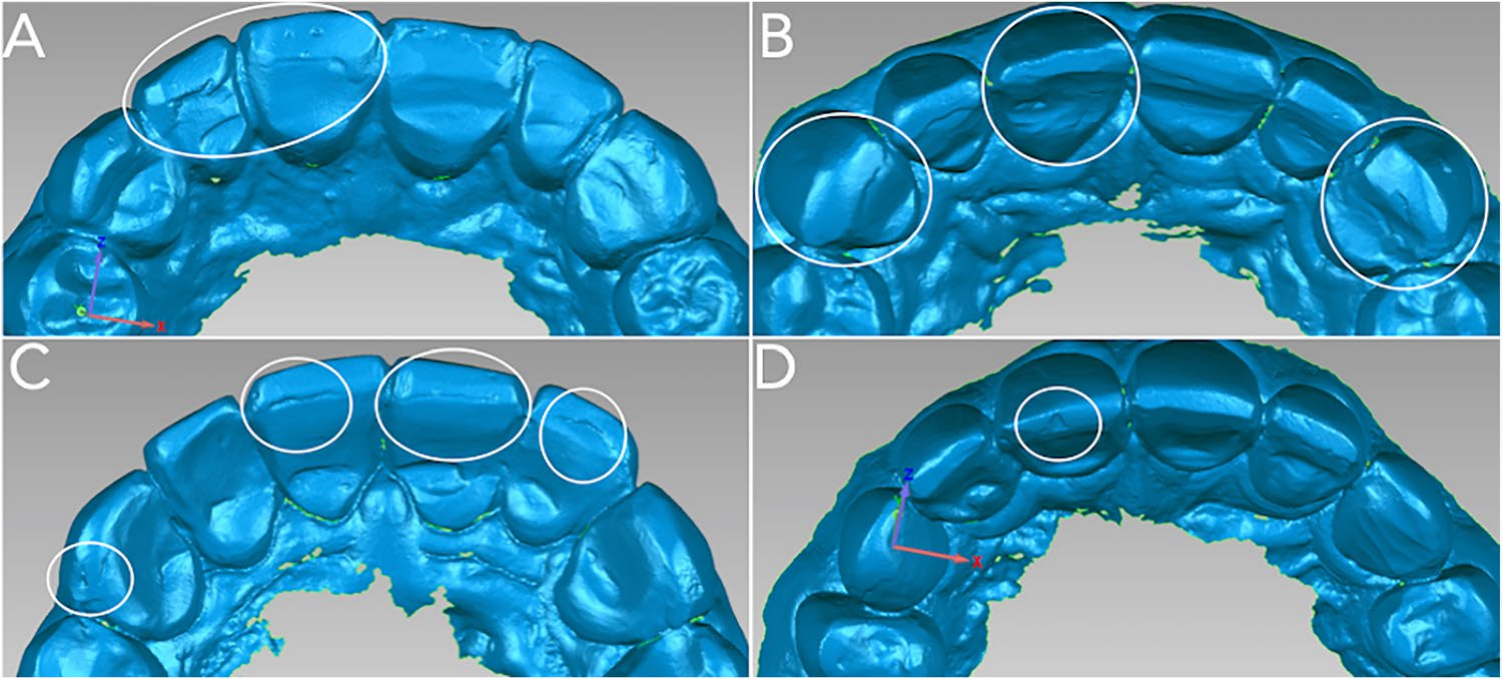
2D images obtained from the 3D scans illustrating the main “degradation features” observed at the 1-month follow-up. Irregularities observed included overhang of the buccal veneer (**A** – teeth 11, 12), surface roughness (**B** – teeth 11, 13, 23), ditching at the interface (**C** – teeth 11, 21, 22), void (**C** – tooth 13), and chip fractures (**D** – tooth 11)

**Fig. 2:**
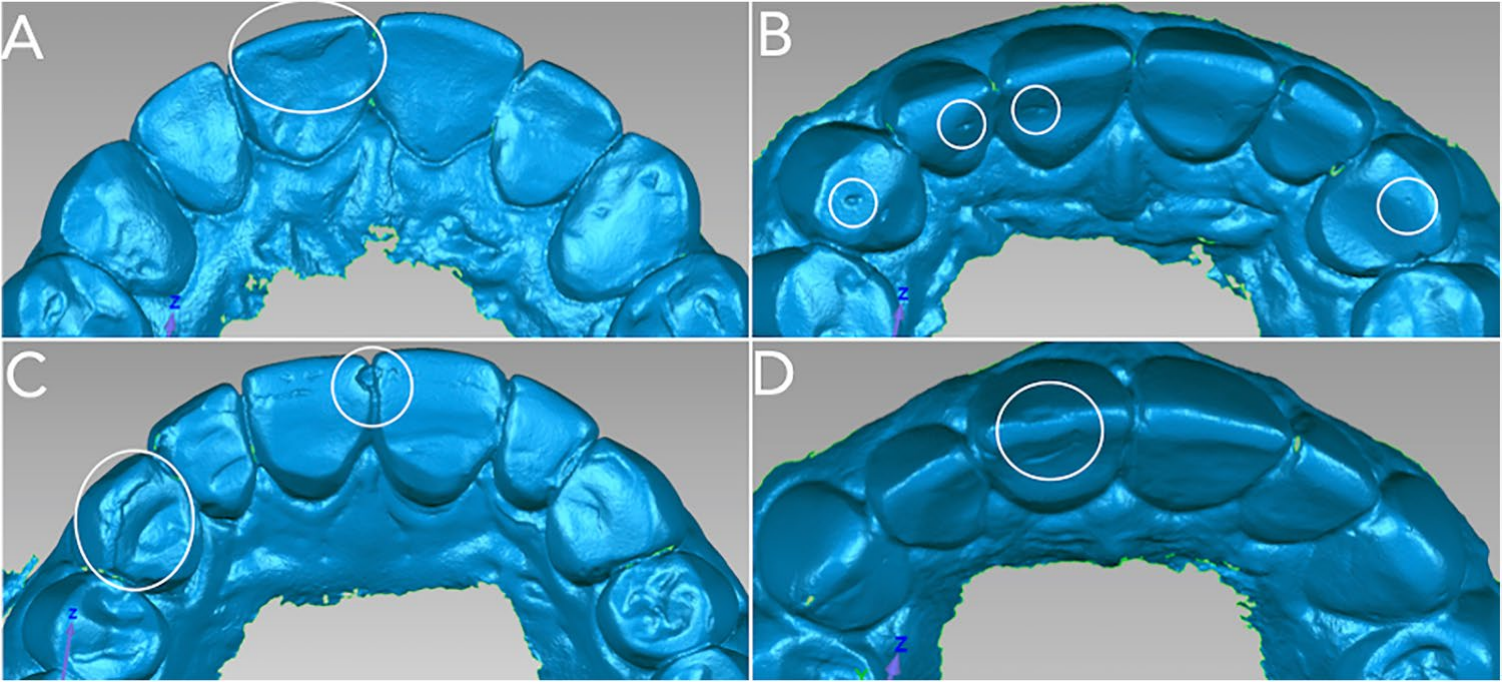
2D images obtained from the 3D scans illustrating the main “degradation features” observed at the 36-month follow-up. Irregularities observed included overhang of the buccal veneer (**A** – tooth 11), voids (**B** – teeth 11, 12, 13, 23), ditching at the interface (**C** – teeth 11, 21), interfacial irregularities (**C** – tooth 13), and fractures involving the buccal veneer (**D** – tooth 11)

The frequency of “degradation features” observed in the restorations is presented in Table 4. Figure 1 (1 month) and Fig. 2 (36 months) present 2D images obtained from the 3D scans illustrating restorations exhibiting these “degradation features.” In the 1-month evaluation, a common observation was the presence of irregularities (41.5%), which encompass aspects such as ridging, overhang of the buccal veneer, surface roughness, and presence of voids (Fig. 1A, B). Ditching was present in 7.4% of restorations in the 1-month evaluation restricted at the interface between the buccal and palatal veneers (Fig. 1C). Fractures, mainly small chips, were present only in 3.7% of the restorations for the same time point (Fig. 1D). The mean and maximum numbers of “degradation features” per patient at 1 month were as follows: irregularities (mean 2.4; max 6), ditching (mean 0.4; max 4), and fracture (mean 0.2; max 2). In the 36-month evaluation, the frequency of irregularities decreased to 37%, with less occurrence of overhang of the buccal veneer and surface roughness (Fig. 2A) and a higher presence of voids (Fig. 2B). Ditching was present in 12.2% (Fig. 2C) and fractures in 10.7% of restorations. The fractures were mainly small chips, but fractures of the buccal veneer were observed in 2.6% of the restorations (Fig. 2D). The mean and maximum numbers of “degradation features” per patient at 36-month were irregularities (mean 2.1; max 5), ditching (mean 0.7; max 4), and fracture (mean 0.6; max 3).

**Table 4:** Frequency and percentage of “degradation features” attributed to restorations after 1 and 36 months of follow-up according to the combination of restorative materials (*n* = 47 patients, number of restorations in each group shown in parenthesis)

Degradation feature		1 month								36 months							
		APX + IPS (*n* = 102)		EST + IPS (*n* = 46)		SUP + SUP (*n* = 80)		LU + SUP (*n* = 42)		APX + IPS (*n* = 102)		EST + IPS (*n* = 46)		SUP + SUP (*n* = 80)		LU + SUP (*n* = 42)	
		Freq	%	Freq	%	Freq	%	Freq	%	Freq	%	Freq	%	Freq	%	Freq	%
*Irregularities*	Present	47	46.1	19	41.3	31	38.8	15	35.7	39	38.2	16	34.8	35	43.8	10	23.8
	Absent	55	53.9	27	58.7	49	61.3	27	64.3	63	61.8	30	65.2	45	56.3	32	76.2
*Ditching*	Present	10	9.8	2	4.3	7	8.8	1	2.4	10	9.8	7	15.2	12	15.0	4	9.5
	Absent	92	90.2	44	95.7	73	91.3	41	97.6	92	90.2	39	84.8	68	85.0	38	90.5
*Fracture*	Present	3	2.9	2	4.3	4	5.0	1	2.4	7	6.9	8	17.4	10	12.5	4	9.5
	Absent	99	97.1	44	95.7	76	95.0	41	97.6	95	93.1	38	82.6	70	87.5	38	90.5

APX + IPS (palatal veneer Clearfil AP-X, buccal veneer IPS Empress Direct), EST + IPS (palatal veneer Clearfil Estenia C&B, buccal veneer IPS Empress Direct), SUP + SUP (palatal and buccal veneers Filtek Supreme XTE), LU + SUP (palatal veneer Lava Ultimate, buccal veneer Filtek Supreme XTE)

**Fig. 3:**
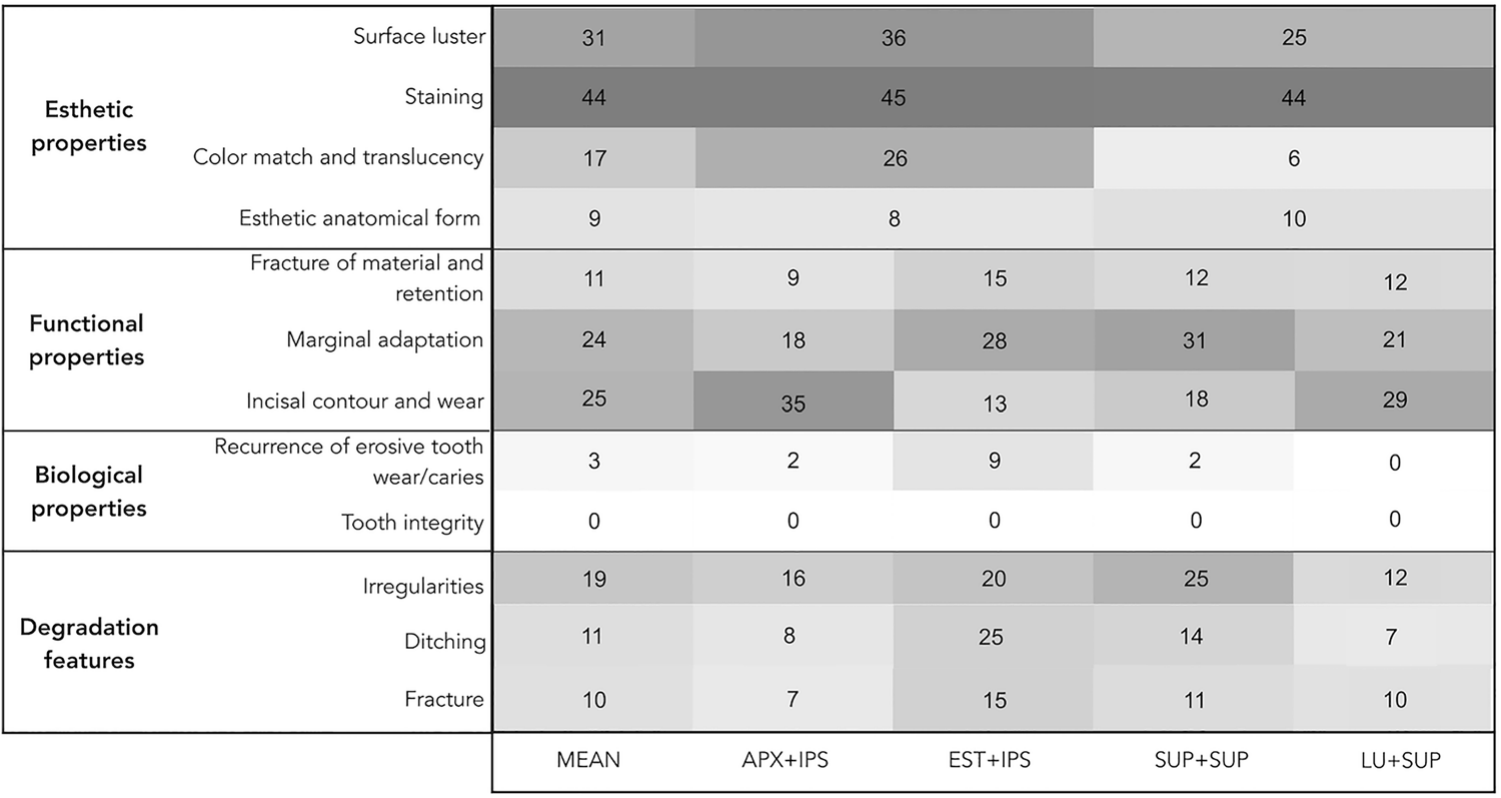
Heatmap showing the percentage of deterioration observed in the restorations after 36 months. Differences in the shades of grey indicate the percentage of deterioration for each criterion, with darker shades of grey representing higher percentages and lighter shades of grey representing lower percentages. Each percentage of deterioration is visible in the respective cell. The FDI criteria (esthetic, functional, and biological properties) and the “degradation features” are presented according to the combination of restorative materials used (*n* = 47 patients, 270 restorations). The overall deterioration is shown in the first column (mean). In the comparison of esthetic properties, groups with similar veneering materials (APX + IPS and EST + IPS; SUP + SUP and LU + SUP) were analyzed together. The same veneering material are merged: APX + IPS and EST + IPS; SUP + SUP and LU + SUP. The combinations of restorative materials are APX + IPS (palatal veneer Clearfil AP-X, buccal veneer IPS Empress Direct), EST + IPS (palatal veneer Clearfil Estenia C&B, buccal veneer IPS Empress Direct), SUP + SUP (palatal and buccal veneers Filtek Supreme), LU + SUP (palatal veneer Lava Ultimate, buccal veneer Filtek Supreme XTE)

Figure 3 shows a graphic representation of the deterioration observed in the restorations after 36 months considering the FDI criteria or “degradation features” and the combination of restorative materials used. The most frequent deterioration in the FDI esthetic properties was for staining (44%) and loss of surface luster (31%). In the functional properties, deterioration was observed mainly regarding incisal contour and wear (25%) and marginal adaptation (24%). Almost no deterioration in the biological properties was observed, with a few exceptions for recurrence of erosive tooth wear/caries after 36 months (3%). With regard to the “degradation features,” the highest percentage of deterioration occurred by the presence of irregularities (19%). The mean and maximum numbers regarding the distribution of deterioration per patient were surface luster (mean 1.8; max 6), staining (2.6; 6), color match and translucency (1; 6), esthetic anatomical form (0.5; 3), fracture of material and retention (0.7; 3), marginal adaptation (1.4; 5), incisal contour and wear (1.4; 6), recurrence of erosive tooth wear (0.2; 4), irregularities (1.1; 5), ditching (0.6; 4), and fracture (0.6; 3).

**Fig. 4:**
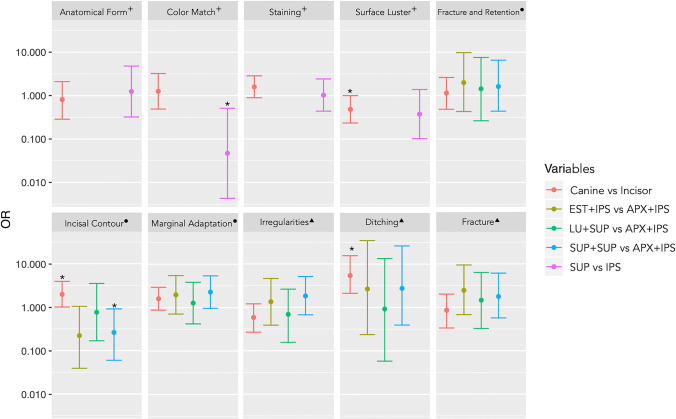
Results for the multiple logistic regression analysis, including odds ratios (OR) and 95% confidence intervals for different variables considering esthetic properties ( +), functional properties (●), and “degradation features” (▲). Asterisks indicate significant associations between the variables and chances of deterioration for a given criterion. In the comparison of esthetic properties, groups with similar veneering materials (APX + IPS and EST + IPS; SUP + SUP and LU + SUP) were analyzed together. The combinations of restorative materials are APX + IPS (palatal veneer Clearfil AP-X, buccal veneer IPS Empress Direct), EST + IPS (palatal veneer Clearfil Estenia C&B, buccal veneer IPS Empress Direct), SUP + SUP (palatal and buccal veneers Filtek Supreme), LU + SUP (palatal veneer Lava Ultimate, buccal veneer Filtek Supreme XTE)

Figure 4 presents results for the multiple logistic regression analysis including the 95% confidence intervals. A significant association was found between canine teeth and a lesser chance of deterioration in surface luster (odds ratio [OR] = 0.482, *p* < 0.05). Buccal veneers made with SUP showed a lesser chance to present deterioration in color match and translucency than buccal veneers made with IPS (OR = 0.047, *p* = 0.01). Canines showed a higher chance of deterioration than incisors in incisal contour and wear [OR = 1.97, *p* < 0.05]. Restorations that combined SUP + SUP showed a lesser chance of deterioration in incisal contour and wear than restorations made with APX + IPS [OR = 0.261, *p* = 0.04]. Canines had 5.5 times more chances of deterioration by ditching than incisors [OR = 5.45, *p* < 0.001]. For the remaining criteria/“degradation features,” no significant associations were observed between the independent variables and deterioration. Recurrence of erosive tooth wear exhibited a very low variation of scores, and tooth wear showed no variation. Thus, biological properties were not included in this analysis.

A descriptive analysis regarding the deterioration observed in restorations according to the number of sessions for placement of the buccal veneers (1 or 2) was carried out. This analysis was restricted to groups in which only direct resin composites were used, i.e., APX + IPS (102 restorations, 47.1% in one session) and SUP + SUP (80 restorations, 55% in one session). For esthetic properties, staining exhibited the highest percentages of deterioration for all materials and sessions, followed by color match and translucency. Regarding the functional properties, restorations with SUP + SUP placed in two sessions had the highest percentage of deterioration in marginal adaptation (47%), whereas restorations with APX + IPS placed in one and two sessions had more deterioration in incisal contour and wear (35% for both). The biological properties were virtually unaffected in the clinical service. When considering the presence of “degradation features,” restorations with SUP + SUP placed in two sessions had the highest percentage of irregularities and ditching (33%).

## Discussion

To our best knowledge, this is the first clinical study to carry out a longitudinal analysis focusing on the deterioration of anterior resin composite restorations placed in patients with moderate to severe tooth wear. To investigate how these restorations deteriorate, clinical data from tooth wear patients including digital photographs and 3D scans were used. To use 3D scans to evaluate restoration deterioration using FDI criteria proved to be an innovative way to score restoration deterioration. Both 3D scans and digital photographs present advantages and limitations, that is why the evaluation combined distinct data assessed from both, providing a complete overview. Overall, deterioration affected up to 44% of restorations, which showed problems mainly regarding surface staining and loss of luster, marginal adaptation, incisal contour, and/or wear after 3 years. We also found that early “degradation features” were detectable in composite restorations placed in this group of patients; irregularities and ditching at the interface between the buccal and palatal veneers were observed after 1 month of clinical service. Thus, the study hypothesis could not be rejected.

The necessity of follow-ups and maintenance is expected for any restoration and patient, but in tooth wear patients the deterioration process might be visible sooner [9, 19]. Deterioration refers to a process in which deleterious changes in the original properties of restorations develop over time [19, 20]. The process may start with less severe clinical events that could allow repair in case of detection before the occurrence of failures [19–23]. If not detected, early deterioration might eventually lead to clinical failures in case even minor irregularities in load-bearing areas, such as the incisal edge or restorative margins, could concentrate mechanical stresses and predispose the occurrence of chippings or larger fractures [21]. Our findings show that the deterioration in anterior restorations placed in worn dentition was mainly detected on esthetic properties after 3 years. Although the FDI scores for most restorations would not indicate the need for replacement, the esthetic problems could be recognized by both the patient and operator and could require intervention [24], reducing success rates. A long-term study on anterior composite restorations showed that repair is a suitable treatment option with benefits over replacement, including the preservation of sound tooth structures and reduced clinical chair time [25].

Patients with worn teeth represent challenging restorative situations because the wear will likely still be taking place after the treatment. As the wear progresses, the irregularities seen in the early stages may be transformed in larger worn areas and wear facets. In this study, a considerable occurrence of irregularities was observed 1 month after placement of the restorations, followed by a decrease in such events after 3 years. A concurrent slight increase in ditching and fractures was observed in the period, suggesting that part of the initial irregularities may have progressed into other deterioration features with time. These findings are in line with those of a clinical study evaluating composite restorations in the mandibular anterior teeth of patients with tooth wear [1]. The authors observed that whereas most restorations exhibited signs of incisal wear in their first few months of service, the progress of wear was minimum afterward. These aspects highlight the importance of establishing realistic restorative expectations when treating the anterior worn dentition with direct or indirect composites. Despite being an effective and conservative approach, composite restorations will most likely suffer from deterioration features and perhaps require checkup appointments more often. The same or an even worse scenario could be expected if ceramic was used as restorative material because of the brittle characteristic of ceramics [22, 26], facilitating crack propagation and restricting plastic deformation.

Another aspect that could affect the longevity of restorations is the presence of an interface between composite layers. Interfaces are considered weak links in adhesive restorations [11, 12]. Any voids or other irregularities along the interface could act as stress magnifiers [27] and result in more deterioration. In the present study, small chippings and ditching at the interface between resin composites were detectable since the early stages of clinical service. This finding indicates that mechanical factors play a significant role in the deterioration of restorations in this group of patients [6, 9, 24, 28] and is congruent with the multifactorial etiology of tooth wear. The high magnitude and cyclic character of loading might develop internal stresses and lead to microcrack propagation within the composite structure, an event that may end up leading to fatigue fractures [21, 29], as manifested by the chippings observed here. The presence of parafunctional habits such as tooth grinding and nail-biting could increase the chances of fracture [30]. In addition, restorations in canines generally had more chances of deterioration than incisors. Canine teeth are subjected to higher occlusal loads than incisors, especially if a canine-guided occlusion is reestablished [31]. The failures are probably a result of the higher mechanical demands resulting in shear stresses within the restoration and at the interfaces. Despite this observation, a canine guide is still recommendable when reestablishing lateral guidance as it is relatively simpler to achieve than group function guidance [32].

One limitation of the present study is that the anterior restorations usually combined more than one type of restorative material, which might imply a heterogeneity of the study and make not possible to distinguish the deterioration occurring at individual materials. However, in order to investigate the deterioration in anterior restorations, the focus should not necessarily be on the materials themselves but on the behavior of composite restorations in this specific group of patients in the course of time. The restorations also included direct and indirect veneers on a same tooth, so it was preferrable to present the results per combination of restorative material instead of pooling the restorations. The only significant association observed for the different combinations of composites was that the group SUP + SUP had a lesser chance of deterioration in incisal contour and wear than the group APX + IPS. This result raises a question about whether the bonding ability between two layers of a same resin composite is better than the bonding between two different materials. In addition, restorations in which the palatal and buccal veneer composites were placed in two sessions showed higher deterioration, in some cases, as compared with single session restorations. This result is in line with those reported recently for patients from the RTWP [7], as we found an increased risk of interventions for two-session restorations. The finding also brings up speculations that the adhesion between two composites placed and cured in the same session might be superior to the adhesion obtained when a “fresh” composite is bonded to an “old” composite, even when the surface is treated with air-abrasion and silane, as it was done in the present study. This topic deserves attention and could be investigated in future laboratory investigations.

The present study provides evidence of the deterioration process taking place in anterior restorations placed in moderate to severe tooth wear patients during a 3-year period of follow-up. In regular patients, clinical failures usually take longer than 5 years to be noticeable [33]. However, the emphasis was not on the overall success of the restorations but on signs of degradation during clinical service, which were noticeable shortly after the restorations were placed and continued to progress over time. For that reason, we presented all signs of degradation observed, including relatively infrequent signs with their respective 95% confidence intervals, to reflect the strength of the observations. The FDI criteria allow detection of early signs of deterioration and/ or failures and to evaluate dental restorations according to categories [34, 35]. Although the clinical examination is indispensable in evaluating restorations, the use of photographs and 3D scans is mentioned as options for calibration in the latest version of the criteria [14]. An interesting aspect of this study was that restorations were assessed retrospectively using a combination between intraoral photographs and 3D scans collected prospectively. Each method may not mean to stand alone and could provide information not aligned to the other, but using both was a strategy to compensate for the limitations of each method.

Previous studies have reported the use of photographs for the assessment of dental restorations. A study that evaluated photographs of posterior composite restorations using the FDI criteria found a high intra-examiner and a slight to fair inter-examiner agreement [36]. Imaging methods, however, may reveal more defects than noticeable in a regular clinical examination. A previous study comparing intraoral photographic and clinical assessments found that images generally provided more defects [37]. Another clinical study using the FDI criteria detected fewer problems in composite restorations by using pictures compared with the direct clinical examination [35]. In the present study, we did not compare the digital evaluation with clinical examination, but the suitability of this method is suggested by the high inter- and intra-examiner agreements achieved in the analysis. As only anterior maxillary restorations were evaluated, esthetic properties are an essential aspect when investigating the deterioration. Therefore, intraoral photographs were used in this study only to assess esthetic properties.

The functional and biological properties and the presence of “degradation features” were assessed only with the 3D scans because they could reveal more information than the photographs. Compared with photographs, the higher contrast of 3D scans may facilitate the visualization of early signs of deterioration. Besides, the scans can be manipulated in x, y, and z directions and zoomed in, providing valuable information of features at surfaces and adhesive interfaces [14]. However, the 3D scans used here were grey-scaled, thus not helpful for esthetic evaluations. To our best knowledge, this is one of the first studies to use 3D scans to assess deterioration and “degradation features” taking place in the clinical service. A recent investigation on tooth wear scores observed higher reliability using gypsum cast records than the 3D scans [38], but also reported higher tooth wear scores on buccal/palatal surfaces when using 3D scans. Another study evaluating a different tooth wear index found a higher detection of initial surface changes using 3D scans than stone casts [39]. The available data on the precision and accuracy of intra-oral scanners indicate that they are precise enough to detect and monitor tooth wear [40–42]. In the present study, data from the 3D scans revealed more defects than intraoral photographs. Digital dentistry is a reality, and the use of 3D scans to evaluate restorations or other intraoral features is likely to increase significantly in the next decade. Currently, it seems that a clinical evaluation of restorations is still necessary, especially when a treatment decision is to be made because the images could increase the risk of overdiagnosis and overtreatment. This is an interesting topic for further studies.

## Conclusions

This longitudinal analysis of anterior resin composite restorations placed in moderate to severe tooth wear patients showed early signs of deterioration after 1 month of clinical service, with a progression of the deterioration over the full study period of 36 months. The deterioration affected mainly the surface of resin composites and adhesive interfaces. A continuous degradation process of anterior restorations may occur when rehabilitating anterior worn teeth with composites, highlighting the need for checkup appointments and the importance of establishing realistic expectations with this group of patients.
